# Blood transfusions increase circulating plasma free hemoglobin levels and plasma nitric oxide consumption: a prospective observational pilot study

**DOI:** 10.1186/cc11359

**Published:** 2012-05-25

**Authors:** Iris C Vermeulen Windsant, Norbert CJ de Wit, Jonas TC Sertorio, Erik AM Beckers, Jose E Tanus-Santos, Michael J Jacobs, Wim A Buurman

**Affiliations:** 1 Department of Surgery, Maastricht University Medical Center, P. Debyelaan 25, PO Box 5800, 6202 AZ Maastricht, the Netherlands; 2 Nutrition and Toxicology Research Institute Maastricht (NUTRIM), Maastricht University Medical Center, P. Debyelaan 25, PO Box 5800, 6202 AZ Maastricht, the Netherlands; 3 Central Diagnostic Laboratory, Maastricht University Medical Center, P. Debyelaan 25, PO Box 5800, 6202 AZ Maastricht, the Netherlands; 4 Department of Pharmacology, Faculty of Medicine of Ribeirao Preto, University of São Paulo, Av. Bandeirantes, 3900 Ribeirão Preto, São Paolo, Brazil; 5 Department of Hematology, Maastricht University Medical Center, P. Debyelaan 25, PO Box 5800, 6202 AZ Maastricht, the Netherlands; 6 Cardiovascular Research Institute Maastricht (CARIM), Maastricht University Medical Center, P. Debyelaan 25, PO Box 5800, 6202 AZ Maastricht, the Netherlands; 7 European Vascular Center Maastricht-Aachen, the Netherlands-Germany University Hospital Aachen, Pauwelsstraße 30, 52074 Aachen, Germany; 8 Department of Vascular Surgery, University Hospital Aachen, Pauwelsstraße 30, 52074 Aachen, Germany

## Abstract

**Introduction:**

The increasing number of reports on the relation between transfusion of stored red blood cells (RBCs) and adverse patient outcome has sparked an intense debate on the benefits and risks of blood transfusions. Meanwhile, the pathophysiological mechanisms underlying this postulated relation remain unclear. The development of hemolysis during storage might contribute to this mechanism by release of free hemoglobin (fHb), a potent nitric oxide (NO) scavenger, which may impair vasodilation and microcirculatory perfusion after transfusion. The objective of this prospective observational pilot study was to establish whether RBC transfusion results in increased circulating fHb levels and plasma NO consumption. In addition, the relation between increased fHb values and circulating haptoglobin, its natural scavenger, was studied.

**Methods:**

Thirty patients electively received 1 stored packed RBC unit (*n *= 8) or 2 stored packed RBC units (*n *= 22). Blood samples were drawn to analyze plasma levels of fHb, haptoglobin, and NO consumption prior to transfusion, and 15, 30, 60 and 120 minutes and 24 hours after transfusion. Differences were compared using Pearson's chi-square test or Fisher's exact test for dichotomous variables, or an independent-sample *t *test or Mann-Whitney U test for continuous data. Continuous, multiple-timepoint data were analyzed using repeated one-way analysis of variance or the Kruskall-Wallis test. Correlations were analyzed using Spearman or Pearson correlation.

**Results:**

Storage duration correlated significantly with fHb concentrations and NO consumption within the storage medium (*r *= 0.51, *P *< 0.001 and *r *= 0.62, *P *= 0.002). fHb also significantly correlated with NO consumption directly (*r *= 0.61, *P *= 0.002). Transfusion of 2 RBC units significantly increased circulating fHb and NO consumption in the recipient (*P *< 0.001 and *P *< 0.05, respectively), in contrast to transfusion of 1 stored RBC unit. Storage duration of the blood products did not correlate with changes in fHb and NO consumption in the recipient. In contrast, pre-transfusion recipient plasma haptoglobin levels inversely influenced post-transfusion fHb concentrations.

**Conclusion:**

These data suggest that RBC transfusion can significantly increase post-transfusion plasma fHb levels and plasma NO consumption in the recipient. This finding may contribute to the potential pathophysiological mechanism underlying the much-discussed adverse relation between blood transfusions and patient outcome. This observation may be of particular importance for patients with substantial transfusion requirements.

## Introduction

Transfusion of stored red blood cells (RBCs) is a common medical procedure, particularly in the context of critical care [1]. Approximately 40% of patients admitted to the ICU receive packed red blood cell (pRBC) transfusion, with a mean of 5 units per patient [2]. The rationale of pRBC administration is enhancement of intravascular oxygen-carrying capacity and thus improvement of tissue oxygenation. However, recent insights into pathological changes of RBC functionality and integrity during storage - collectively known as the storage lesion - has drawn attention to the potential negative consequences of pRBC transfusion as it limits the post-transfusion survival of RBCs *in vivo *[3]. The storage lesion includes decreased RBC viability and deformability, increased RBC aggregability and adhesiveness, enhanced susceptibility to oxidative damage, formation of membrane vesicles resulting in loss of surface area, and increased cell density [3-5]. Furthermore, several changes to the RBC microenvironment (the storage medium) have been reported; a decrease in pH; accumulation of proinflammatory substances, hemoglobin-derived free iron, and microvesicles containing large amounts of free hemoglobin (fHb) [6]; and increased levels of hemolysis markers such as potassium, arginase-1, and fHb in the storage medium [3,7-9]. Generally, increasing storage duration aggravates the storage lesion [3].

The profound beneficial effects of transfusion cannot be understated. Nevertheless, the number of studies reporting on the potential adverse effects of (massive) pRBC transfusion on patient morbidity and mortality has increased significantly in recent years, which has initiated an intense debate on the benefits and risks of pRBC, particularly focusing on the effects of younger versus older pRBCs [10-15]. Although the exact mechanisms underlying the adverse effects of pRBC administration have not yet been fully elucidated, the release of fHb and its influence on the intravascular nitric oxide (NO) metabolism after transfusion has been attributed an important role [6]. fHb was found to be a potent scavenger of NO, the most important endogenous vasodilator [16]. In line with this finding, increased fHb levels in patients with chronic and acute hemolysis have been associated with decreased NO bioavailability within the microcapillary bed, decreased organ perfusion and increased organ injury [16-18]. Similarly, transfusion of stored pRBC units may enhance fHb concentrations in patients after transfusion - for instance, as a consequence of premature intravascular rupture of the transfused erythrocytes, or due to the transfusion of the fHb-containing storage medium [8]. In line with this hypothesis, it was recently shown that transfusion of fHb-containing supernatant of stored erythrocytes in rats caused a significant increase in blood pressure that correlated with the fHb levels in the supernatant [6].

This prospective observation pilot study aimed to investigate whether transfusion of 1 or 2 pRBC units can result in enhanced circulating fHb levels and plasma NO consumption in the recipient. In addition, we studied whether the pRBC storage duration or pre-transfusion levels of plasma haptoglobin (Hp) - the physiological fHb scavenger - influenced the change of plasma fHb in the recipient after transfusion.

## Materials and methods

### Patients

Based on previous experiments, we calculated that inclusion of 22 patients would provide sufficient power (0.80 with α = 0.05) to detect significant increases in plasma fHb levels in the recipient after transfusion of 2 stored pRBC units. To compare patients receiving 1 versus 2 stored RBC units, all patients screened during the inclusion period receiving only 1 pRBC unit were also included. To that end, 58 patients admitted to the Department of Hematology and Oncology of the Maastricht University Medical Center between 1 May 2010 and 1 November 2010 were screened for eligibility for study participation using predetermined inclusion and exclusion criteria. This resulted in the inclusion of 22 patients undergoing transfusion with 2 pRBC units, and eight patients undergoing transfusion with 1 pRBC unit. All patients provided written informed consent at least 24 hours before transfusion. The study protocol was approved by the Ethical Review Board of the Maastricht University Medical Center in accordance with the Declaration of Helsinki (amended in Seoul, 2008).

We only included adult patients (≥ 18 years) with normal kidney function and normal liver function, who were in a stable clinical phase of their disease. The rationale of choosing patients with a (hematological) malignancy was that these patients frequently need blood transfusions. Furthermore, these patients generally have a central venous line (in the jugular or subclavian vein) enabling nontraumatic blood sampling, and thus minimizing sampling-induced hemolysis. Absence of such a central venous line was a reason for exclusion. Other inclusion criteria were normal kidney function defined as an estimated glomerular filtration rate of 60 ml/minute/1.73 m^2 ^or higher, assessed by the abbreviated Modification of Diet in Renal Disease recommended by the National Kidney Foundation [19]. Normal liver function was indicated by normal pre-transfusion bilirubin levels. Furthermore, presence of a central venous line (in the jugular or subclavian vein) was mandatory to enable nontraumatic blood sampling, thus minimizing sampling-induced hemolysis.

C-reactive protein levels exceeding 10 mg/l were a reason for patient exclusion since an active inflammatory process may lead to increased Hp levels. Hp is the physiological fHb scavenger, so patients with increased Hp concentrations may be able to clear fHb more readily compared with patients with normal plasma Hp levels (0.25 to 1.9 g/l). Because Hp is not routinely measured in patients prior to blood transfusion at our hospital, we were not able to use Hp concentrations directly to include or exclude patients. Other exclusion criteria included pre-existent hemolytic disease, and transfusion of pRBCs within 72 hours prior to inclusion. We chose a timeframe of 72 hours on the basis of previous (unpublished) data showing that, even after strong increases of plasma fHb, these levels are back to normal values at 24 hours after peak fHb levels were observed (in a stable clinical condition without ongoing hemolysis). The investigators did not have any influence on the selection of the transfused pRBCs with regard to storage duration or any other parameter.

### Red blood cell products

All transfused pRBCs were collected and prepared for transfusion by the Sanquin Blood Supply Foundation, the Netherlands. The maximum storage duration of pRBC units allowed in the Netherlands after the collection date is 35 days. All pRBC units were pre-storage leucodepleted, according to the standard protocol. The storage solution consisted of a mixture of saline, adenine, glucose and mannitol. The mean volume and hematocrit level of the transfused pRBC units were 275 ml and 0.60, respectively. The pRBCs were subsequently distributed to and stored by the Central Diagnostic Laboratory of the Maastricht University Medical Center until use. In cases where 2 pRBC units were administered, the storage duration of both pRBC units was never more than 2 days apart.

The transfusion protocol used at our institution was the nationally used transfusion protocol as defined by the Dutch Institute for Healthcare Improvement CBO (Central Accompaniment Organization). In short, patients and donors are matched based on blood type (A, B, AB, and O) and rhesus D phenotype. Patients and donor blood were also matched according to the presence of irregular antibodies (or autoantibodies). In women under the age of 45, donor blood was always Kell-negative. The majority of transfused pRBCs were of blood type O (*n *= 32, 61.5%) and were Rh-negative (*n *= 33, 63.5%). The remainder of pRBC units were either of blood type A (*n *= 16, 30.8%) or blood type B (*n *= 4, 7.7%). Eight patients were transfused with irradiated (25 Gy) pRBCs. Each pRBC unit was administered in a 60-minute to 90-minute timeframe. None of the patients received fresh frozen plasma or platelets during the study period.

### Blood sampling and sample processing

Central venous blood was drawn from the venous line by an experienced nurse at six preset time points; pre-transfusion (blood sample taken on the morning of transfusion in the context of routine patient care), 15 minutes after transfusion (T15) of all pRBCs (1 or 2 units), 30 minutes after transfusion (T30), 60 minutes after transfusion (T60), 120 minutes after transfusion (T120), and the morning after transfusion (T24). Prior to every sample collection, the central venous line was flushed with sterile saline to prevent sampling of the previously administered pRBCs (which was given via the same line) or remnants of the previously collected blood sample. Whole blood was gently collected using sterile syringes, instead of standard blood collection tubes (vacutainers), to prevent vacuum-induced hemolysis. After collection of two syringes, the blood from the second syringe (not contaminated with sterile saline) was immediately transferred to an ethylenediamine-tetraacetic acid-containing blood collection tube without vacuum (Becton Dickinson, Franklin Lakes, NJ, USA), carefully mixed by gentle rotation, and subsequently stored for a maximum of 1 hour at 4°C until further processing. After centrifugation (1,500 × *g *at 4°C for 15 minutes without braking), plasma was aliquoted and stored at -20°C until further analysis.

### Laboratory analysis

#### Free hemoglobin

The fHb concentrations, indicating hemolysis, were measured in all patient plasma samples (*n *= 30) and in the storage medium of every administered pRBC (*n *= 52). fHb was analyzed by derivative spectrometry as described in more detail elsewhere [20]. The detection limit of this assay was 2 μmol/l.

#### Haptoglobin

Plasma Hp concentrations of all patient samples and Hp levels in the storage medium of all transfused pRBC were measured on a validated Beckman LX20 clinical chemistry analyzer (Beckman Coulter, Brea, CA, USA) via a turbidimetric method by the Laboratory of Hematology of the Maastricht University Medical Center.

#### Nitric oxide consumption assay

To evaluate whether increased fHb levels influenced the NO-consuming capacity of plasma after transfusion, we analyzed the NO consumption in all plasma samples of 13 randomly selected patients from our total patient group of 30 (40%; four patients transfused with 1 pRBC unit, nine patients transfused with 2 pRBC units), and in the storage medium of the 22 pRBC units administered to these patients. Random selection of patients was carried out using SPSS software (SPSS Inc., Chicago, IL, USA). Unfortunately, we were not able to analyze every sample from the total patient group due to time and cost constraints.

The complete protocol of the NO consumption assay is described in more detail elsewhere [16,21]. In short, a 40 μM solution of the NO donor (DETA NONOate; Cayman Chemical, Ann Harbor, MI, USA) was prepared in PBS (pH 7.4) in a glass vessel purged with nitrogen in line with a NO chemiluminescence analyzer (Sievers Model 280i; GE, Boulder, CO, USA). The subsequent decay of DETA NONOate, releasing NO, produced a steady-state NO signal of about 50 to 70 mV. When the signal became stable, 50 μl plasma samples or standards were injected into the DETA NONOate solution, decreasing the NO signal in cases of NO consumption. Data were transferred to the software program ORIGIN Version 6.1 (OriginLab, Northampton, MA, USA) for analysis of the area under the curve of decreasing NO signal over time. The amount of NO consumption by plasma is quantified by comparison of the area under the curve with that of NO gas standards (produced from injections of nitrite into tri-iodide).

### Statistical analysis

Continuous data in tables are presented as the median and interquartile range (25th to 75th percentile), and dichotomous data as *n *(%). Continuous data presented in figures are depicted as the mean ± standard error of the mean. Differences in patient characteristics between study groups were compared using Pearson's chi-square test for dichotomous variables, with Fisher's correction when appropriate, and using the independent-sample *t *test or Mann-Whitney U test for continuous data depending on the Gaussian distribution (checked using histograms and normal Q-Q plots). Continuous data with multiple timepoints were analyzed using repeated one-way analysis of variance with Bonferroni *post-hoc *correction or the Kruskall-Wallis test with Dunn's *post-hoc *correction, depending on the Gaussian distribution. Correlations were analyzed using Spearman correlation (indicated as *R*_s_) or Pearson correlation (*r*) depending on the Gaussian distribution. Statistical calculations were made using SPSS 15.0 for Windows (SPSS Inc.), and Prism 4.03 for Windows (GraphPad Software Inc., San Diego, CA, USA). *P *< 0.05 was considered to indicate statistical significance.

## Results

Thirty patients were prospectively studied. The median age of the total patient group was 56 years and the majority of patients was male (*n *= 25, 83.3%). Most patients had been diagnosed with acute myeloid leukemia (*n *= 21, 70%) and all patients were in a stable clinical phase of their disease. Eight patients (26.7%) were transfused with 1 pRBC unit, and 22 patients (73.3%) with 2 pRBC units. Interestingly, the increase in hemoglobin following transfusion was similar in patients receiving 1 or 2 pRBC units: from 8.2 g/l to 9.7 g/l. Other baseline and pRBC unit characteristics also did not statistically differ between the two groups (Table 1).

**Table 1:** Patient and packed red blood cell unit characteristics

	Patients receiving 1 pRBC unit (*n *= 8)	Patients receiving 2 pRBC units (*n *= 22)	*P *value
Patient characteristics			
Age (years)	51.0 (23.0-61.0)	58.5 (43.5-68.0)	0.118
Male	87.5 (7)	81.8 (18)	1.000
Disease			0.530
Acute myeloid leukemia	75.0 (6)	68.2 (15)	
Multiple myeloma	12.5 (1)	-	
Burkitt's lymphoma	-	4.5 (1)	
Acute lymphoblastic leukemia	-	4.5 (1)	
Testis carcinoma	12.5 (1)	-	
Myeloid sarcoma	-	4.5 (1)	
Non-Hodgkin B-cell lymphoma	-	9.1 (2)	
Myelodysplastic lymphoma	-	9.1 (2)	
Pre-transfusion hemoglobin (g/dl)	8.2 (7.8-8.5)	8.2 (7.5-8.4)	0.383
Post-transfusion hemoglobin (g/dl)	9.7 (9.0-10.0)	9.7 (9.2-10.2)	0.459
Pre-transfusion plasma haptoglobin (g/l)	2.1 (1.3-3.8)	2.4 (1.5-2.8)	0.784
Pre-transfusion serum bilirubin (μmol/l)	16.8 (12.2-28.0)	15.0 (13.4-22.3)	0.872
Pre-transfusion serum AST (IU/l)	16.0 (14.0-44.0)	14.0 (11.0-21.0)	0.231
Pre-transfusion serum ALT (IU/l)	28.0 (17.0-50.0)	24.5 (14.8-35.3)	0.507
Pre-transfusion serum creatinine (mmol/l)	65.0 (57.0-75.0)	67.0 (58.3-85.5)	0.508
Packed red blood cell characteristics			
Blood type			1.000
O	75.0 (6)	59.1 (13)	
A	25.0 (2)	31.8 (7)	
B	-	9.1 (2)	
AB	-	-	
Rh-negative	62.5 (5)	59.1 (13)	1.000

Data presented as median (interquartile range, 25% to 75%) or percentage (*n*). pRBC, packed red blood cell. There were no significant changes between the study groups for any of the variables shown.

### Prolonged storage of pRBCs results in elevated fHb levels and increased NO-consuming capacity of the storage medium

**Figure 1:**
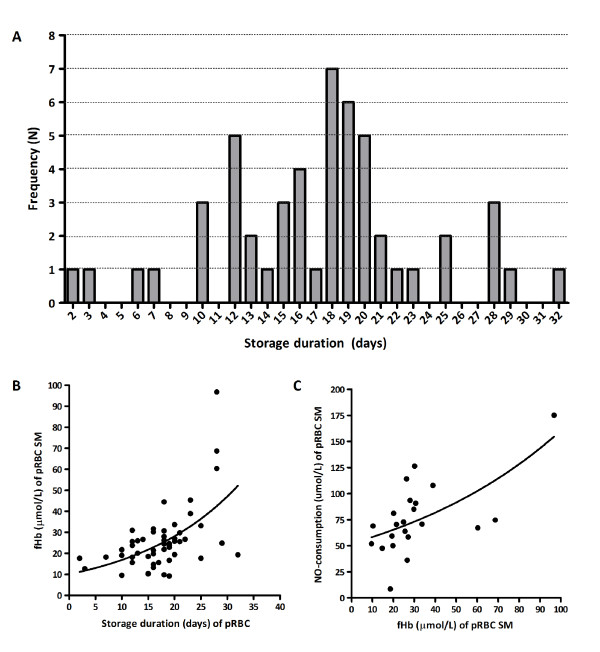
**Packed red blood cell storage duration correlates with increased free hemoglobin and nitric oxide consumption**. **(A) **Histogram of storage duration for all transfused packed red blood cell (pRBC) units (*n *= 52). Mean storage duration was 17.2 days (range 2 to 32 days). **(B) **Correlation between storage duration and the free hemoglobin (fHb) concentrations of the storage medium (SM) for each pRBC unit (*n *= 52). **(C) **Correlation between the fHb concentration and nitric oxide (NO) consumption within the SM in a random selection of 22 pRBC units. Black lines depict a nonlinear regression (exponential) curve.

In total, 52 pRBC units were administered. The mean storage duration of the pRBC units was 17.2 days (range 2 to 32 days; Figure 1A), which was similar to previous reports [12]. Of all transfused units, 4 pRBC units (7.2%) were younger than 1 week while 11 pRBC units (21.2%) had been stored for 3 weeks or longer. To analyze the degree of hemolysis within the pRBC during storage, we measured fHb concentrations in the storage medium of each transfused unit. The mean fHb level of the storage medium was 25.9 ± 2.2 μmol/l, with a peak level of 96.7 μmol/l. Corresponding to previous reports [6,8], prolonged storage duration correlated with fHb concentrations within the storage medium (*r *= 0.51, *P *< 0.001; Figure 1B). As fHb is a potent NO scavenger, we hypothesized that fHb concentrations within the storage medium would positively correlate with the level of NO consumption of the storage medium. The NO consumption of the storage medium averaged 76.0 μmol/l (range 8.6 to 175.1 μmol/l), and was significantly correlated with fHb levels of the storage medium (*r *= 0.61, *P *= 0.002; Figure 1C) and with the storage duration of the pRBCs (*r *= 0.62, *P *= 0.002; data not shown).

### Transfusion of 2 pRBC units results in increased plasma fHb concentrations and NO consumption in patients

**Figure 2:**
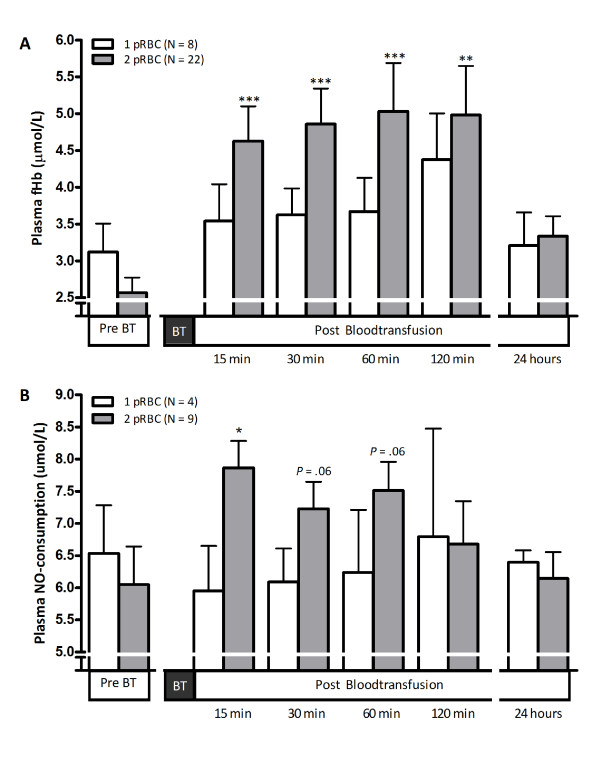
**Transfusion of stored red blood cells significantly increases plasma free hemoglobin and nitric oxide consumption**. **(A) **Course of plasma free hemoglobin (fHb) in patients receiving 1 (white bars) or 2 (grey bars) packed red blood cell (pRBC) units from baseline levels before blood transfusion (BT) to 15, 30, 60 and 120 minutes and 24 hours after BT. ***Significant changes of fHb levels within groups compared with baseline fHb concentrations; *P *< 0.001. **(B) **Course of plasma nitric oxide (NO) consumption in a random selection of four patients transfused with 1 pRBC unit and nine patients transfused with 2 pRBC units. *Significant differences of fHb levels within groups compared with baseline fHb concentrations; *P *< 0.05.

We next studied the effect of pRBC transfusion on plasma fHb levels of the 30 recipients. Figure 2A depicts the baseline and post-transfusion fHb concentrations, grouped according to transfusion of 1 or 2 pRBC units. This subdivision was made based on the notion that the latter group would (theoretically) be subjected to a higher fHb load and could thus display higher plasma fHb values after transfusion. Baseline plasma fHb levels were statistically similar between both groups. Interestingly, plasma fHb values increased significantly in patients receiving 2 pRBC units compared with baseline concentrations as early as 15 minutes after transfusion (from 2.6 ± 0.2 μmol/l to 4.6 ± 0.5 μmol/l, respectively; *P *< 0.001), and fHb levels remained significantly enhanced compared with baseline values throughout the next 2 hours. After 24 hours, fHb levels had returned to pre-transfusion levels. Plasma fHb concentrations in patients transfused with 1 pRBC unit did not change significantly over time. Both the storage duration and fHb concentrations of the storage medium of the administered pRBCs were similar between patients transfused with 1 or 2 pRBC units (*P *= 0.63 and *P *= 0.97, respectively), and it is therefore less likely that these factors confound the observed differences in plasma fHb concentrations after transfusion between both groups.

Irradiation of stored pRBCs has been reported to increase the storage lesion, especially membrane permeability [7]. To exclude the potential confounding effect of pRBC irradiation on post-transfusion fHb concentrations in patients, we compared fHb concentrations in the storage medium of irradiated (*n *= 13) and nonirradiated (*n *= 39) pRBC units - which were 23.6 ± 2.7 μmol/l and 26.7 ± 2.7 μmol/l, respectively (*P *= 0.97). Moreover, post-transfusion fHb concentrations in plasma were not significantly different between patients receiving irradiated blood products versus patients receiving nonirradiated blood products (*P *> 0.05 on all timepoints; data not shown).

NO consumption was subsequently measured in all plasma samples of a randomly selected subgroup of 13 patients (Figure 2B). In line with the fHb data, the plasma NO consumption rose significantly in patients receiving 2 pRBC units from 6.1 ± 1.5 μmol/l at baseline to 7.9 ± 1.3 μmol/l 15 minutes after transfusion (*P *= 0.03), remaining elevated during the first hour after transfusion (*P *= 0.06). Plasma NO consumption did not significantly change in patients receiving 1 pRBC unit. Overall, NO consumption in plasma correlated significantly with plasma fHb concentrations (*R*_s _= 0.35, *P *= 0.003; data not shown).

Summarizing, the storage medium of stored pRBC units is a potent NO-consuming medium as it contains high levels of fHb. In line with this finding, plasma levels of fHb and NO consumption increased significantly in patients after transfusion of 2 pRBC units.

### Pre-transfusion haptoglobin levels influence post-transfusion plasma fHb concentrations in patients

The current debate concerning the assumed negative effect of transfusion of pRBC units on patient outcome mainly focuses on storage-related factors such as storage duration. As we found a significant effect of storage duration on fHb levels and NO consumption within the storage medium, we studied whether the storage duration of the pRBC units influenced post-transfusion fHb concentrations *in vivo*. Surprisingly, in our study the storage duration of the pRBC unit did not correlate with post-transfusion fHb concentrations or NO consumption in the recipient, even after correction for the number of pRBCs transfused and for fHb concentrations of the storage medium (data not shown).

**Figure 3:**
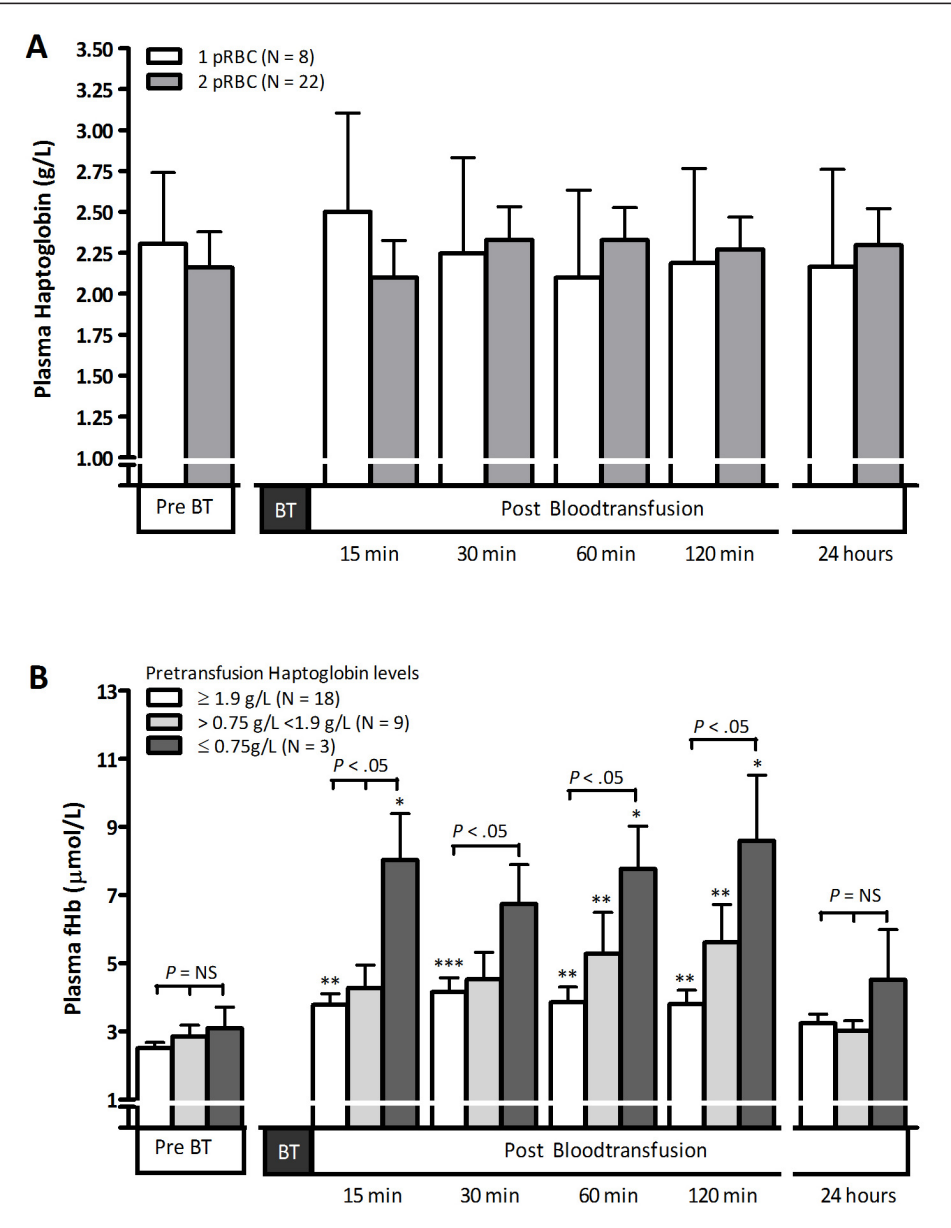
**Post-transfusion plasma haptoglobin, and pre-transfusion haptoglobin impact on post-transfusion free hemoglobin and nitric oxide consumption**. (**A**) Change of plasma haptoglobin in patients receiving 1 (white bars) or 2 (grey bars) packed red blood cell (pRBC) units from levels before blood transfusion (BT) to 15, 30, 60 and 120 minutes and 24 hours after BT. (**B**) Change of plasma free hemoglobin (fHb) in patients with pre-transfusion haptoglobin plasma concentrations of 1.9 g/l or higher (white bars), between 0.75 and 1.9 g/l (light-grey bars), or 0.75 g/l or lower (dark-grey bars). Significant changes of fHb levels within groups compared with baseline (pre-transfusion) levels: *P < 0.05, **P < 0.01, ***P < 0.001. Statistical differences between groups are indicated with black bars and according P values. NS, nonsignificant.

We next studied the effect of pRBC transfusion on post-transfusion Hp concentrations in the recipient. As Hp is the physiological scavenger protein for fHb, and considering the significant increase of circulating fHb concentrations following transfusion of 2 pRBC units, we expected decreased post-transfusion Hp levels in these patients. However, Hp levels did not change significantly after transfusion in patients receiving 1 pRBC unit or 2 pRBC units (Figure 3A).

We subsequently investigated whether pre-transfusion Hp concentrations of the recipient influenced post-transfusion fHb levels, as patients with low pre-transfusion Hp levels may have a lower capacity to clear fHb. To that end, we divided patients into three groups based on their pre-transfusion plasma Hp level. Eighteen patients (60%) displayed high pre-transfusion Hp concentrations exceeding 1.9 g/l (upper normal level). In contrast, three patients (10%) presented Hp concentrations below 0.75 g/l. Post-transfusion fHb concentrations showed a more pronounced increase from pre-transfusion levels in patients with the lowest pre-transfusion Hp concentrations, compared with patients with higher pre-transfusion Hp levels (*P *< 0.05; Figure 3B). Storage duration of the administered pRBCs was similar between the three groups (*P *= 0.20), as was the mean number of pRBCs transfused (1 or 2 units, *P *= 0.85) and the mean fHb concentration of the storage medium of the pRBC units (*P *= 0.53). Furthermore, similar results were obtained when patients receiving 1 or 2 pRBC units were analyzed separately (data not shown). Unfortunately, the study was not sufficiently powered to allow statistical analysis of changes in post-transfusion plasma NO consumption based on pre-transfusion Hp levels.

## Discussion

The relation between transfusion of stored blood and adverse outcome in patients is the subject of intense debate. Although the development of functional and structural changes of the RBCs during storage is undisputed, it remains controversial whether these changes carry any clinical relevance. In recent years, several studies have reported on the association between increased storage duration of pRBCs (reflecting increased storage lesion) and adverse outcome in trauma patients [22], septic patients [13,23], the critically ill [24], and patients undergoing cardiac surgery [12,25]. In contrast, other investigators did not find such associations [14,15,26,27] The development of hemolysis within the pRBC unit, with release of the NO-scavenging protein fHb, may contribute to the potential adverse relation between blood transfusions and patient outcome [6].

This observational study investigated whether transfusion of 1 or 2 stored pRBC units can result in increased fHb concentrations and plasma NO consumption in the recipient, and whether the increase in fHb levels is dependent on the storage duration of the pRBC unit(s) or on pre-transfusion recipient Hp levels. To this end, we studied patients with a (hematological) malignancy in a stable clinical phase of their disease as these patients could be used as a model to study changes of plasma markers before and after blood transfusions. First, we demonstrated that prolonged storage significantly increased fHb concentrations and NO consumption within the pRBC storage medium, which was in line with previous reports [8,28]. Second, we were able to demonstrate for the first time that transfusion of 2 pRBC units significantly enhanced plasma fHb concentrations and NO consumption in patients. Third, the increase in post-transfusion fHb concentrations was most profound in patients with low pre-transfusion Hp levels and was absent in patients with highest Hp concentrations, irrespective of the number of transfused pRBCs, the storage duration of the transfused units, and the fHb concentrations within the storage medium.

To date, studies reporting on the postulated adverse effects of pRBC transfusion on patient outcome principally focused on the contribution of pRBC-related factors, with most attention being paid to the effect of storage duration. We could not confirm a correlation between prolonged storage and increased fHb levels (or increased NO consumption) in our patients. Perhaps the uneven distribution of the storage duration of the pRBC units and the relatively small number of patients prevented us from finding such a relation. Interestingly, however, we were able to show an effect of pre-transfusion plasma Hp levels of the recipient on changes of fHb following transfusion. High plasma Hp levels prior to transfusion may improve the physiological buffer capacity of the recipient to increased plasma fHb concentrations. One could even hypothesize that patients displaying increased Hp levels, such as patients with profound systemic inflammation [29,30], may be better protected against the potential adverse effects of transfused fHb. In contrast, patients undergoing (complex) cardiovascular surgery could be particularly sensitive to transfusion-induced, fHb-mediated NO consumption, as these patients frequently suffer Hp depletion during surgery [31].

Our data may be of significant importance for patients who require massive transfusion, such as critical care patients, trauma patients, or patients undergoing major aortic, cardiac, orthopedic, or gynecologic surgery [18,32]. Transfusion of multiple pRBC units may result in a significant increase of plasma fHb levels and NO consumption. Since plasma fHb concentrations of 6 μmol/l have been shown to significantly impair the NO metabolism *in vivo *[16], and levels over 10 μmol/l have been linked to the development of renal injury and renal dysfunction [18], excessive pRBC transfusion may potentially decrease microcirculatory blood flow and contribute to organ compromise in these patients. Indeed, transfusion of pRBCs to treat anemia during cardiopulmonary bypass has been shown to be independently associated with increased urinary markers of intestinal damage, renal injury, and deterioration of kidney function compared with untreated anemic patients [33,34]. A similar independent association between pRBC transfusions and worse outcome was found in anemic critically ill patients [35].

Although the presented associations between the number of transfused pRBC units and increased levels of fHb and increased NO consumption do not imply causality, the results of the current study elicit an interesting dilemma regarding blood transfusions. On the one hand, refraining from transfusing a (seriously) anemic patient might further impair blood oxygen-carrying capacity and tissue oxygenation, possibly causing tissue ischemia and organ failure. On the other hand, (massive) transfusion of pRBC units, containing high amounts of fHb, could significantly increase plasma fHb concentrations and NO consumption in the recipient. This could hamper microcirculatory blood flow and may also ultimately lead to cellular damage and organ failure. As the beneficial effects of blood transfusions are undisputed, and safe alternatives to blood storage are still lacking, efforts should be made to minimize the unwanted effects of stored RBC transfusion. If our results are confirmed by larger studies, the current study provides opportunities for the development of preventive or treatment strategies.

Although we were able to demonstrate significant results, the relatively small patient population prevented correction for confounding variables such as disease severity. Future studies are therefore essential to provide additional insight into the (causal) relationships between transfusion of stored blood, increased plasma fHb concentrations and plasma NO consumption, decreased microcirculatory perfusion, and clinical outcome of the recipient. Furthermore, we did not study the contribution of RBC microvesicles, known to be released during hemolysis and known to contain high concentrations of fHb, on post-transfusion fHb concentrations in the recipient [36]. In addition, the role of increased free (nontransferrin-bound) iron levels was not studied [37]. Lastly, we acknowledge the fact that we extrapolate data and results obtained in a specific patient group to other patient settings. Notwithstanding, we consider that the results of this pilot study provide an important additional insight into the much-discussed relationship between stored blood transfusion and adverse outcome in patients, and therefore warrant additional investigation.

## Conclusion

Our data indicate that transfusion of 2 stored RBC units significantly increases plasma fHb concentrations and NO consumption in the recipient. These data are of interest in light of the ongoing debate and evaluation of the proposed negative association between pRBC transfusions and patient outcome, and may serve as a tool to improve patient morbidity and mortality after transfusion.

## Key messages

• Prolonged storage of pRBC units results in increased fHb concentrations and NO consumption within the storage medium.

• Transfusion of 2 pRBC units, in contrast to transfusion of 1 pRBC unit, significantly increases circulating fHb levels and plasma NO consumption in the recipient, irrespective of storage duration.

• High pre-transfusion plasma Hp levels may be protective as they are inversely related to post-transfusion fHb concentrations in the recipient.

## Abbreviations

fHb: free hemoglobin; Hp: haptoglobin; NO: nitric oxide; PBS: phosphate-buffered saline; pRBC: packed red blood cell; RBC: red blood cell.

## Competing interests

The authors declare that they have no competing interests.

## Authors' contributions

ICVW designed and performed the research, collected, analyzed, and interpreted data, and drafted and approved the manuscript. NCJdW performed the research, interpreted data, and drafted and approved the manuscript. JTCS performed the research, analyzed and interpreted data, and drafted and approved the manuscript. EAMB interpreted data and drafted and approved the manuscript. JET-S performed the research, analyzed and interpreted data, and drafted and approved the manuscript. MJJ drafted and approved the manuscript. WAB designed the study, interpreted data, and drafted and approved the manuscript. All authors have read and approved the manuscript for publication.
